# Ocular Toxicity Profile of Targeted Cancer Therapy (TCT) at a US Tertiary Cancer Center

**DOI:** 10.7759/cureus.40597

**Published:** 2023-06-18

**Authors:** Moe Ameri, Nagham Al Zubidi, Azadeh Razmandi, Andrew Whyte, Aung Naing, Nimisha A Patel, Dan S Gombos

**Affiliations:** 1 Internal Medicine, University of Texas Medical Branch at Galveston, Galveston, USA; 2 Investigational Cancer Therapeutics / Division of Cancer Medicine, University of Texas MD Anderson Cancer Center, Houston, USA; 3 Ophthalmology, University of Texas MD Anderson Cancer Center, Houston, USA; 4 Head and Neck Surgery, University of Texas MD Anderson Cancer Center, Houston, USA; 5 Investigational Cancer Therapeutics, University of Texas MD Anderson Cancer Center, Houston, USA

**Keywords:** immunotherapies, ocular iraes, ocular toxicity, immune-related adverse events (iraes), targeted cancer therapy

## Abstract

Purpose: Targeted cancer therapy (TCT) is a significant advancement in oncology with promising survival improvement in patients with cancer and remarkable effects on various cancers. There is evidence suggesting a connection between specific TCT classes and the occurrence of immune-related adverse events (irAEs). Our study aims to investigate the potential ocular toxicities of different classes of TCT, provide a better understanding of these toxicities, and aid in the future development of screening and management recommendations for ocular irAEs.

Design: Retrospective observational case series.

Participants: Only ocular immune-related AEs were included in the study; patients on TCT who received a new ophthalmic diagnosis were seen at the MD Anderson Cancer Center.

Methods: Between 2010 and 2019, we retrospectively reviewed the medical records of 6,354 patients on TCT at a large US tertiary cancer center.

Results: The criteria for data analysis were met by 1861 patients. TCT was associated with a wide range of class-specific ocular irAEs. There was a statistically significant correlation between ocular toxicity with polytherapy with a p-value of <0.001. Furthermore, there was a statistically significant correlation between toxicity and BRAF, epidermal growth factor receptor (EGFR), and ICI <0.001, <0.001, and 0.006, respectively.

Conclusion: Our cohort is the most extensive case series in English literature, demonstrating the increased risk of class-specific ocular toxicity associated with TCT, which sheds some light on the importance of developing standardized grading criteria and management guidelines.

## Introduction

Targeted cancer therapy (TCT) is an exceptional cancer treatment that influences the immune system, blocks various pathways in cancer cell development, and interferes with specific signaling and angiogenesis pathways [1]. Through the years, the number of Food and Drug Administration (FDA)-approved and off-label indications have expanded with promising survival benefits in various cancers, e.g., metastatic melanoma, renal cell carcinoma (RCC), non-small cell lung cancer (NSCLC), Hodgkin lymphoma, and other malignancies. TCT is an exceptional cancer treatment that influences the immune system, blocks various pathways in cancer cell development, and interferes with specific signaling and angiogenesis pathways [1]. In theory, TCTs are more tumor-targeted and less destructive than standard chemotherapy. However, some TCT classes have been linked to unfavorable side effects known as immune-related adverse events (irAEs) [2]. 

Ocular irAEs are one of the most common side effects of TCT. They are becoming more crucial in managing patients taking these medications but have not received sufficient attention. The eye's susceptibility to ocular irAEs can be attributed to its unique microenvironment. Factors such as the delicate balance of growth factors and cell receptors, the highly specialized structure with extensive genetic expression, the presence of cancer-promoting signaling molecules, and the capacity for vascular formation collectively contribute to this vulnerability. These characteristics make the eye a distinct and intricate setting where the interplay of various mechanisms puts it at risk of experiencing these irAEs [3-4].

The two major TCT classes are small compounds and monoclonal antibodies. Monoclonal antibodies (MAbs) are synthetic macromolecules designed to target specific cell surface antigens via two mechanisms: stimulatory by agonistic cell protection and inhibitory work at immunological synapses by facilitating the immune system's detection of cancer cells. Monoclonal antibodies include immune checkpoint inhibitors (ICI), anti-programmed cell death protein-1 (PD-1) agents (such as nivolumab and pembrolizumab), anti-programmed death ligand-1 (PDL1) agents (such as atezolizumab, avelumab, and durvalumab), and anticytotoxic T lymphocyte-associated protein 4 (CTLA-4) agents (ipilimumab). Epidermal growth factor receptor (EGFR) inhibitors, (such as Cetuximab, Panitumumab, Erlotinib, and Gefitinib), work by focusing on and obstructing the signaling pathway and signal transduction that promote tumor growth. This suppresses cell proliferation and causes apoptosis [5]. HER2 inhibitors (Trastuzumab, Ado-trastuzumab, Emtansine) inhibit the oncogenic properties of HER2 receptors in HER2-positive cancers and stimulate immune responses against HER2-amplified cancers [6].

Small molecules (-nibs) are synthetic compounds that block extracellular or intracellular enzymes, including tyrosine kinase inhibitors (TKIs), BRAF inhibitors (vemurafenib, dabrafenib), mitogen-activated protein/extracellular signal-regulated kinase (MEK) inhibitors (trametinib, cobimetinib), and fibroblast growth factor receptor (FGFR) inhibitors (erdafitinib and pemigatinib) [7-15].

Our study aims to highlight the broad spectrum of ocular irAEs, their type, frequency, and severity, recognize class-specific TCT that present with severe complications, and provide a better understanding of these side effects. Future research is required to guide the monitoring and management of these irAEs.

## Materials and methods

Retrospectively, we reviewed the medical records of 6,354 patients on TCT between January 2010 and December 2019, who presented to the ophthalmology clinic, underwent an ophthalmologist examination, and received a new ophthalmic diagnosis at our institution MD Anderson Cancer Center (MDACC).

Patients with pre-existing ocular pathology before starting TCT were excluded because it was a possible confounding risk factor. This study was approved by MD Anderson Cancer Center's institutional review board (IRB). Thirty-four TCTs were identified and classified into nine classes: EGFR inhibitors, HER2 inhibitors, BRAF inhibitors, MEK inhibitors, FGFR inhibitors, ICI, ALK, TKI, and enfortumab vedotin-ejfv. Table 1 outlines the two main types of TCT.

**Table 1:** The main classes of TCT. TCT, targeted cancer therapy

TCT	Mechanism of action	Agent(s)
Monoclonal antibodies	Synthetic biomolecules (-mabs)	Anticytotoxic T-lymphocyte-associated protein 4 (CTLA-4) agents (ipilimumab) anti-programmed cell death protein-1 (PD-1) (nivolumab, pembrolizumab) anti-programmed death ligand-1 (PDL1) (atezolizumab, avelumab, durvalumab)
Small molecules	Synthetic molecules (-nibs)	Tyrosine kinase inhibitors (imatinib, ceftinib, erlotinib) BRAF inhibitors (vemurafenib, dabrafenib) mitogen-activated protein/extracellular signal-regulated kinase (MEK) inhibitors (trametinib, cobimetinib)

Data from the nine classes of TCT were analyzed to determine the ocular irAEs. irAEs were grouped into different structural categories: external adnexa: lid, lash, glands, peri-ocular, dry eye, cornea, conjunctiva, episclera, and sclera, anterior and posterior uvea, pupils, lens, optic nerve, retina, choroid, vitreous are separate entities besides accommodation and refraction abnormalities. We reported the demographics, including age and gender, presenting symptoms, cancer diagnosis, TCT used frequency, the severity of ocular irAEs, time to presentation, management, and clinical outcome. The categorical variables studied were presented as counts and row percentages. The severity of ocular events is graded by the standard terminology criteria of adverse events (CTCAE) grades (Version 4.0 and 5.0) [16]. The CTCAE system includes five grades based on the severity of signs and symptoms [16]. Each has a corresponding visual acuity and intervention guidelines. Grade I has mild to no symptoms; Grade II has moderate symptoms; Grade III has severe or medically significant adverse events; however, it is not immediately life-threatening for Grade IV toxicities that may be life or sight-threatening. Urgent intervention required for Grade V involves a death related to the adverse event, as shown in Table 2. Statistical analysis was done using multiple comparisons via Kruskal-Wallis. Using the Cochran-Armitage and Fisher's Exact tests, we reported the clinical patterns associated with the increasing severity of irAEs. The statistical significance was defined as p < 0.05.

**Table 2:** Common terminology criteria for adverse events (CTCAE) grades (versions 4.0 and 5.0). VA, visual acuity; BCVA, best corrected visual acuity

Grade 1	Grade 2	Grade 3	Grade 4	Grade 5
Mild	Moderate	Severe	Sight-threatening Life-threatening	Death
Asymptomatic or mild symptoms BCVA 20/20 Intervention not indicated.	Symptomatic with a moderate decrease in VA BCVA >20/40 or <3 Lines of decreased vision from baseline. Medical intervention indicated	Symptomatic with a marked decrease in VA BCVA < 20/40 or >3 lines of decreased vision from baseline (up to 20/200). Invasive intervention indicated	Life-threatening consequences BCVA < 20/200 Urgent intervention indicated	Death

## Results

We identified 6,354 patients on TCT out of 22,726 patients referred for clinical research encounters in the ophthalmology clinic between January 2010 and December 2019; of the 6,354 patients, 1861 patients met the criteria for data analysis. Nine hundred twenty (49.44%) were females, and 941 (50.56%) were males. Their ages ranged from 6.6 to 99.3 years, with a mean of 62.5 years and a standard deviation of 14.11 years. The average time of developing ocular toxicity was six months after starting TCT. The minimum time was three days, while the maximum was 40 months.

There were 33 patients (1.77%) on ALK inhibitors, 219 pts (11.77 %) on BRAF inhibitors, 248 pts (13.32 %) on EGFR inhibitors, 104 pts (5.59 %) on FGFR inhibitors, 160 pts (8.60%) on HER2 inhibitors, 1100 pts (59.10%) on ICPI, 376 pts (20.20%) on MEKI, and 206 pts (11.07%) on TKI. Most of the patients, 1442 (77.49%), were on one agent, but 419 pts (22.51%) were on more than one agent: 278 pts (14.94%) on two agents, 125 (6.72%) on three agents, and 16 (0.9%) on four agents. 

The study examined the correlations between various treatments and ocular adverse events. No statistically significant correlations were observed with the use of ALK, BRAF, FGFR, HER2, ICI, MEKI, or TKI inhibitors. However, a significant correlation was found with the use of EGFR inhibitors, indicating a higher likelihood of experiencing conjunctivitis and scleritis in 18.41% of patients (37/201) (p = 0.008, odds ratio = 5.65, 95% CI = 1.76-18.19). Additionally, dry eye syndrome was observed in 34.35% of patients (271/789), and cataracts were observed in 3.38% of patients (13/385). Significant correlations were identified with the use of BRAF inhibitors (p = 0.010, odds ratio = 1.81, 95% CI = 1.14-2.87), EGFR inhibitors (p < 0.001, odds ratio = 2.90, 95% CI = 1.87-4.48), HER2 inhibitors (p = 0.006, odds ratio = 1.82, 95% CI = 1.18-2.81), and ICI (p = 0.002, odds ratio = 0.63, 95% CI = 0.47-0.84). These results suggest an increased likelihood of developing these specific adverse events with the use of these treatments.

Retina and choroidal abnormalities were found in 26/222 pts (11.71%). There was a statistically significant correlation with MEK inhibitors (p =0.008, odds ratio = 3.00, 95% CI = 1.30-6.91). Uveitis was noted in 36/66 pts (54.55%). Statistically significant correlations were found with BRAF inhibitors (p < 0.001, odds ratio = 8.13, 95% CI = 2.35-28.11), MEK inhibitors (p = 0.009, odds ratio = 5.00, 95% CI = 1.56-15.97), and TKI (p = 0.007, odds ratio not calculable). Subjective visual disturbances were observed in 182/946 pts (19.24%).

There were statistically significant correlations with the use of BRAF inhibitors (p = 0.026, odds ratio = 1.62, 95% CI = 1.06-2.48), EGFR inhibitors (p < 0.001, odds ratio = 2.84, 95% CI = 1.85-4.36), MEK inhibitors (p = 0.035. odds ratio = 1.50, 95% CI = 1.03-2.19), and TKI (p = 0.003, odds ratio = 2.02, 95% CI = 1.27-3.22). Agent ICI was not significantly correlated with subjective visual disturbances (p = 0.05, odds ratio = 0.72, 95% CI = 0.52-1.00). At least one toxicity was found in 580/1861 patients (31.17%).

There were statistically significant correlations for finding any toxicity with BRAF inhibitors (p < 0.001, odds ratio = 1.87, 95% CI = 1.40-2.49), EGFR inhibitors (p < 0.001, odds ratio = 1.94, 95% CI = 1.48-2.55), HER2 inhibitors (p = 0.007, odds ratio = 1.57, 95% CI = 1.13-2.20), and ICI (p = 0.006, odds ratio = 0.76, 95% CI = 0.62-0.93). Findings of external adnexa disorders in 19/442 pts (4.30%), glaucoma in 44/226 pts (19.47%), optic nerve disorders in 15/216 pts (6.94%), and pupil and accommodation abnormalities in 9 /192 pts (4.69%) not statistically significantly correlated with the use of any of the therapeutic agents or possible confounding variables.

Possible correlations of confounder variables, including gender, age group, and multiple agents administered, were assessed via univariate analysis. There were no statistically significant correlations of any ocular toxicity with gender. There were statistically significant correlations between oldest (>= 72.8 years) vs. youngest (<= 53.4 years) age quartiles and findings of dry eye (p < 0.001, odds ratio = 7.06, 95% CI = 4.42-11.28) or any ocular toxicity (p < 0.001, odds ratio = 1.69, 95% CI = 1.28-2.24). Administering more than one agent to a patient was statistically significantly correlated with findings of dry eye (p < 0.001, odds ratio = 1.92, 95% CI = 1.36-2.71), subjective visual disturbances (p < 0.001, odds ratio = 2.30, 95% CI = 1.61-3.27), and any toxicity (p < 0.001, odds ratio = 1.67, 95% CI + 1.33-2.10). As shown in Table 3, the age quartile was also statistically significantly correlated with the administration of EGFR inhibitors (p = 0.026), FGFR inhibitors (p < 0.001), HER2 inhibitors (p < 0.001), ICI (p < 0.001), and TKI (p = 0.002). The administration of multiple agents correlated with the administration of BRAF inhibitors (p < 0.001), EGFR inhibitors (p < 0.001), HER2 inhibitors (p < 0.001), ICI (p < 0.001), and MEK inhibitors (p < 0.001).

In multivariate analysis to assess whether models contain the agents (TCT) and the confounding factors, young-old quartile age, and several agents, Table 3 showed the agents to be significantly correlated with the incidence of dry eye, subjective visual disturbances, and finding of any toxicity; HER2 inhibitors were still significantly correlated with findings of dry eye (p < 0.001, odds ratio = 5.41, 95% CI = 2.56-11.36), as was ICI (p < 0.001, odds ratio = 2.74, 95% CI = 1.60-4.70). For subjective visual disturbances, only TKI significantly correlated (p = 0.049, odds ratio = 1.98, 95% CI = 1.00-3.91). For finding any toxicity, BRAF inhibitors (p = 0.039, odds ratio = 1.70, 95% CI + 1.03-2.82), EGFR inhibitors (p = 0.017, odds ratio = 1.62, 95% CI = 1.09-2.42), HER2 inhibitors (p < 0.001), odds ratio = 2.40, 95% CI = 1.47-3.92), and ICI (p = 0.024, odds ratio = 1.40, 95% CI = 1.04-1.88) was still statistically significantly correlated.

**Table 3:** Multivariate analysis to assess models containing the agents (TCT) as well as the confounding factors young-old quartile age, and number of agents. TCT, targeted cancer therapy

Agent or factor		Dry eye		Subjective visual disturbance		Any toxicity found	
		p-Value	Odd ratio (95% Cl)	p-Value	Odd ratio (95% Cl)	p-Value	Odd ratio (95% Cl)
BRAF	# Agent given	0.147	1.58 (0.85–2.92)	0.018	2.43 (1.16–5.08)	0.356	1.21 (0.80–1.85)
	Young-old age quartile	<0.001	6.94 (4.33–11.11)	0.071	1.53 (0.96–2.42)	<0.001	1.69 (1.28–2.25)
EGFR	# Agent given	0.051	1.69 (1.00–2.86)	0.053	1.68 (0.99–2.85)	0.020	1.48 (1.06–2.06)
	Young-old age quartile	<0.001	6.80 (4.22–10.99)	0.065	1.54 (0.97–2.45)	0.001	1.64 (1.24–2.18)
HER2	# Agent given	0.006	2.19 (1.25–3.83)	N/A	N/A	0.001	1.73 (1.25–2.40)
	Young-old age quartile	<0.001	10.10 (5.93–17.54)	N/A	N/A	<0.001	1.83 (1.37–2.44)
ICPI	# Agent given	0.005	2.22 (1.27–3.88)	0.009	1.97 (1.18–3.30)	0.002	1.68 (1.21–2.34)
	Young-old age quartile	<0.001	9.90 (5.81–16.95)	0.031	1.69 (1.05–2.72)	<0.001	1.81 (1.35–2.41)
MEK	# Agent given	N/A	N/A	0.041	2.35 (1.04–5.35)	N/A	N/A
	Young-old age quartile	N/A	N/A	0.069	1.53 (0.97–2.42)	N/A	N/A
TKI	# Agent given	N/A	N/A	0.011	1.93 (1.12–3.23)	N/A	N/A
	Young-old age quartile	N/A	N/A	0.043	1.62 (1.02–2.58)	N/A	N/A

The most often reported symptoms were subjective visual disturbances and impaired vision. Our analysis also revealed that most irAEs were grade I and were treated topically with preservative-free artificial tears or by observation. In grades II and IV, patients needed topical, local, or systemic corticosteroids. In 30% of the cases, the severity of toxicity reached grade III levels, leading to the discontinuation or temporary suspension of TCT treatments. In 70% of grade IV, grade III-IV toxicity was associated with systemic toxicities requiring stopping the treatment. Some grade IV toxicity required surgical procedures such as pars plana vitrectomy. 

## Discussion

The list of FDA-approved and off-label indications for TCT has grown over the years. These agents have demonstrated a survival benefit in many cancers; however, the extent of TCT ocular toxicity is still unknown. This study aims to explore the potential ocular toxicities of various classes of TCT and determine which TCT needs close monitoring by an ophthalmologist to provide better knowledge of these ocular toxicities necessary for developing screening and management recommendations.

Our cohort is the most extensive case series in English literature that reviewed 34 agents and provides a comprehensive agent-specific set of ocular toxicities associated with and highlighted the increased potential of class-specific ocular toxicity. Ocular irAEs can affect any ocular structure. Thus, it is critical to consider irAEs when assessing patients on TCT.

Our data analysis showed variability in the onset of TCT ocular toxicity, with an average time of 6 months ranging from 3 days to a maximum of 40 months. In addition, our study demonstrated that agent-specific and ocular structure-specific ocular irAEs make it very important to consider when starting a patient on treatment with TCT. A significant correlation was found between EGFR inhibitors and cataract formation; our data corroborate a prior study that revealed that EGF and EGFR signaling promotes the development of epithelial-mesenchymal transition (EMT) and regulate lens epithelial cells (LECs) through a miR-26b-dependent mechanism. These findings show that EGF and EGFR signaling drove MYC expression via attracting HDAC3, protooncogene MYC overexpression blocked miR-26b, leading to the production and acceleration of the development of EMT. Based on these findings, it is proposed that targeting EGFR could serve as a potential therapeutic approach for the treatment of posterior capsule opacity (PCO). This is in contrast to recent studies that have indicated the potential of EGF to enhance TGF2 activity, leading to an increase in epithelial-to-mesenchymal transition (EMT) in lens epithelial cells (LECs). It is hypothesized that the role of EGFR signaling in cataract development should be considered, and it is suggested that by directly reducing EGFR signaling, both EGF and TGF2 activity can be simultaneously diminished, offering a possible avenue for cataract prevention [17-18].

Our data also showed that ICI significantly correlates with the development of conjunctivitis. In previous studies, conjunctivitis with ICI presented with irritation and conjunctival injection, with severe and refractory cases being reported. Most documented cases were of sterile conjunctivitis, which responded well to topical steroid therapy, none of which required ICI suspension [19-20].

Numerous ocular tissues have been identified to display high levels of PD-L1, which may be essential in avoiding autoimmunity. Dry eye syndrome was the most common irAEs reported (34.35%). Uveitis was the most often reported irAEs associated with BRAF inhibitors in literature. Our data demonstrated a statistically significant association between BRAF inhibitors, especially dabrafenib, with panuveitis, anterior uveitis, and overall ciliary body and iris anomalies.

On the other hand, clinical studies and post-marketing surveillance showed retinal damage and "MEK inhibitors associated retinopathy" as the most often reported irAEs. In our study, MEK inhibitors as monotherapy or combination therapy showed a statistically significant correlation with retinal and choroid abnormalities, corroborating prior literature [21]. These retinal abnormalities were found to occur as early as days or weeks after initiation of treatment and resolve after discontinuation [22]. Therefore, we might speculate that these abnormalities are likely class-specific consequences of BRAF and MEK inhibitors as monotherapy or combination.

Numerous investigations have demonstrated that BRAF inhibitors as monotherapy were linked to medication resistance due to the reactivation of the MAPK (mitogen-activated protein kinase) pathway. To counteract the paradoxical stimulation of the MAPK pathway, combination therapy with BRAF and MEK inhibitors is frequently employed. Studies have revealed that combination therapy has synergistic anti-cancer actions. However, the discussion of combination therapy causing an increased risk of toxicity is still controversial due to much disagreement among authors [22-25]. Our data showed that combination therapy strongly correlates with increases in the iris, ciliary body, retinal, and choroid abnormalities. Serous retinal detachment was the most frequent irAEs associated with combination therapy.

Our study showed a statistically significant correlation between combination therapy or multiple therapies with BRAF, EGFR, HER2, ICI, and MEK inhibitors use; any toxicity compared to monotherapy suggests an additive effect of combination therapy [22-28]. Due to the anticipated rise in the use and indications of TCT, it is crucial for practicing ophthalmologists to be aware of and recognize the symptoms of potential ocular toxicity, especially given that ocular TCT-related irAEs may be challenging to distinguish from the disease's direct impact or unrelated consequences. Recognizing and differentiating these complications is imperative to the proper care and treatment of the patient. In addition, conducting ophthalmological baseline examinations before treatment may assist in detecting any pre-existing ocular conditions and may help reduce ocular side effects from treatment.

Our study is the most extensive in the English literature, with a large cohort from one institution with 34 TCTs aiding in quantifying TCT's ocular adverse events, which can better inform ophthalmologists about these risks without data from extensive epidemiologic studies. Our findings also highlight the importance of adverse event awareness and reporting, particularly by specialists such as ophthalmologists, to allow for the best characterization, targeted clinical surveillance, and early treatment initiation to improve visual outcomes. 

Limitations

Our study limitation includes those with any retrospective study, the overlap between the different diagnostic categories, and the overlaps between different presentations, including under-reporting and over-reporting. However, we do acknowledge that, as with any observational study, residual confounders cannot be ruled out. We could not precisely categorize signs and symptoms due to the uncertainty and non-specificity of some terminology and anatomical diagnoses. 

Future direction

This report generates a hypothesis that serves as a starting point for further research of these TCTs and the pathophysiology of ocular damage. Future clinical trials are required to improve the classification and reporting of irAEs, severity, and treatment logarithm. Our team is working to study each TCT group independently with a closer focus on the class-specific irAEs, grading of irAEs, and building a logarithm for treatment. 

## Conclusions

In this study, we present the largest case series available in the English ophthalmic literature, focusing on evaluating and characterizing ocular irAEs associated with specific TCTs at a prominent tertiary cancer center in the United States. Our findings underscore the unique nature of ocular irAEs, which often exhibit side effects specific to their respective therapeutic classes. It is crucial for ophthalmologists to identify these ocular toxicities to prevent irreversible damage promptly. Additionally, further research is warranted to develop guidelines for screening, monitoring, and effective management of these ocular complications.
